# Rapid shifts in the age-specific burden of malaria following successful control interventions in four regions of Uganda

**DOI:** 10.1186/s12936-020-03196-7

**Published:** 2020-03-30

**Authors:** Simon P. Kigozi, Ruth N. Kigozi, Adrienne Epstein, Arthur Mpimbaza, Asadu Sserwanga, Adoke Yeka, Joaniter I. Nankabirwa, Katherine Halliday, Rachel L. Pullan, Damian Rutazaana, Catherine M. Sebuguzi, Jimmy Opigo, Moses R. Kamya, Sarah G. Staedke, Grant Dorsey, Bryan Greenhouse, Isabel Rodriguez-Barraquer

**Affiliations:** 1 Department of Disease Control, London School of Hygiene & Tropical Medicine, Keppel Street, London, WC1E 7HT UK; 2 Infectious Diseases Research Collaboration, PO Box 7475, Kampala, Uganda; 3 USAID’s Malaria Action Program for Districts, PO Box 8045, Kampala, Uganda; 4 Department of Epidemiology & Biostatistics, University of California, San Francisco, 550 16th Street, San Francisco, CA 94158 USA; 5 Child Health and Development Centre, Makerere University College of Health Sciences, Mulago Hospital Complex, PO Box 7072, Kampala, Uganda; 6 School of Public Health, Makerere University College of Health Sciences, Mulago Hospital Complex, PO Box 7072, Kampala, Uganda; 7 School of Medicine, Makerere University College of Health Sciences, Mulago Hospital Complex, PO Box 7072, Kampala, Uganda; 8 National Malaria Control Division, Uganda Ministry of Health, Kampala, Uganda; 9 Department of Clinical Research, London School of Hygiene & Tropical Medicine, Keppel Street, London, WC1E 7HT UK; 10 Department of Medicine, University of California, San Francisco, 1001 Potrero Ave, SFGH Building 30, San Francisco, CA 94110 USA; 11 Division of HIV, ID, and Global Medicine, University of California, San Francisco, 1001 Potrero Ave, SFGH, Building 3, San Francisco, CA 94110 USA; 12 Chan Zuckerberg Biohub, San Francisco, CA USA

**Keywords:** Malaria, Routine surveillance, Age distribution, Reduced transmission, Burden shift

## Abstract

**Background:**

Malaria control using long-lasting insecticidal nets (LLINs) and indoor residual spraying of insecticide (IRS) has been associated with reduced transmission throughout Africa. However, the impact of transmission reduction on the age distribution of malaria cases remains unclear.

**Methods:**

Over a 10-year period (January 2009 to July 2018), outpatient surveillance data from four health facilities in Uganda were used to estimate the impact of control interventions on temporal changes in the age distribution of malaria cases using multinomial regression. Interventions included mass distribution of LLINs at all sites and IRS at two sites.

**Results:**

Overall, 896,550 patient visits were included in the study; 211,632 aged < 5 years, 171,166 aged 5–15 years and 513,752 > 15 years. Over time, the age distribution of patients not suspected of malaria and those malaria negative either declined or remained the same across all sites. In contrast, the age distribution of suspected and confirmed malaria cases increased across all four sites. In the two LLINs-only sites, the proportion of malaria cases in < 5 years decreased from 31 to 16% and 35 to 25%, respectively. In the two sites receiving LLINs plus IRS, these proportions decreased from 58 to 30% and 64 to 47%, respectively. Similarly, in the LLINs-only sites, the proportion of malaria cases > 15 years increased from 40 to 61% and 29 to 39%, respectively. In the sites receiving LLINs plus IRS, these proportions increased from 19 to 44% and 18 to 31%, respectively.

**Conclusions:**

These findings demonstrate a shift in the burden of malaria from younger to older individuals following implementation of successful control interventions, which has important implications for malaria prevention, surveillance, case management and control strategies.

## Background

In Africa, the burden of malaria has decreased significantly, primarily through the scale-up of vector control interventions, including long-lasting insecticidal nets (LLINs), and indoor residual spraying of insecticide (IRS) [1–3]. These interventions were coupled with improved case management using artemisinin-based combination therapy (ACT), and intermittent preventive therapy with sulfadoxine-pyrimethamine for pregnant women [4, 5]. Whereas the impact of malaria control interventions is generally measured in terms of changes in *Plasmodium falciparum* infection prevalence and case numbers [1], more evidence is needed on how interventions influence the age distribution of malaria cases, a vital marker of progress in malaria control [6]. In high transmission settings, younger children bear the brunt of the malaria burden [7, 8], particularly for severe malaria and malaria deaths [9, 10]. However, it is unclear how quickly and to what extent the age distribution of uncomplicated malaria cases may shift with changes in transmission, following the successful implementation of control interventions.

Malaria surveillance efforts have generally focused on children under 5 years of age, a group that contributes the majority of reported cases [11] and are thus the focus of control measures and research, in areas of stable malaria transmission in sub-Saharan Africa. School-aged children (5–15 years) have received less attention due to the lower morbidity and occurrence of severe outcomes in this group [12]. However, older children often experience low-density asymptomatic infections and have been identified as important contributors to the infection reservoir for onward transmission [13]. Even less attention is given to adults (over 15 years), except for pregnant women [14], despite documented high prevalence of asymptomatic infections with high parasitaemia in adults [15, 16].

Commitments to malaria control made at the Abuja Summit in 2000 [17] led to World Health Organization (WHO) recommendations of universal coverage with LLINs, beginning in 2007 [18]. In Uganda, access to LLINs increased gradually, first among pregnant women and children under 5 years of age [19, 20]. This later culminated in universal LLIN campaigns aimed at one LLIN for every two household residents [21], with nationwide distributions in 2013–2014 [22] and 2017–2018. Furthermore, IRS was implemented in 10 districts effective 2010, then shifted to 14 districts from 2014 onwards [23, 24]. In northern Uganda, integrated community case management (iCCM) of malaria began from 2016 to 2017 and the 10 districts plus one also resumed IRS [25].

Following the scale-up of malaria control efforts in Uganda, confirmed malaria cases reported from health facilities declined by an average of 10.8% per year between 2013 and 2015 before increasing in 2016 [26]. Moreover, malaria indicator surveys (MIS) showed that parasite prevalence in children under 5 years of age declined from 42% in 2009 to 19% in 2014–2015 [27, 28], whilst malaria mortality reportedly decreased from 59 to 23 deaths per 100,000 between 2010 and 2017 [25].

This study aimed to investigate the impact of control interventions on the age distribution of malaria cases, using high-quality malaria surveillance data from four sites in Uganda, where mass LLIN distribution was conducted at all four sites and IRS implemented at two, one of which also received iCCM.

## Methods

### Malaria surveillance

The National Malaria Control Division (NMCD) of the Ministry of Health has conducted surveillance through the health management information system (HMIS) since 2007. However, more detailed malaria surveillance including collection of individual-level data has been conducted in selected malaria reference centres (MRCs) since 2006. MRCs are level III or IV health facilities located across the country, representing varied transmission settings as previously described [29, 30]. In summary, patients visiting these centres are assessed at the outpatient department (OPD) and basic information recorded using an OPD registry, including history of fever, age, gender, malaria diagnostic test results, and treatments prescribed. Individual visit-level OPD records are then entered into an MS Access database (Microsoft Corporation, Redmond, WA, USA) and sent to the Uganda Malaria Surveillance Programme (UMSP) data centre and cleaned before transfer to STATA (Stata Corporation, College Station, TX, USA) for analysis. Data from these sites are used to generate monthly reports that are shared and reviewed by the NMCD and other stakeholders.

### Study sites

**Fig. 1:**
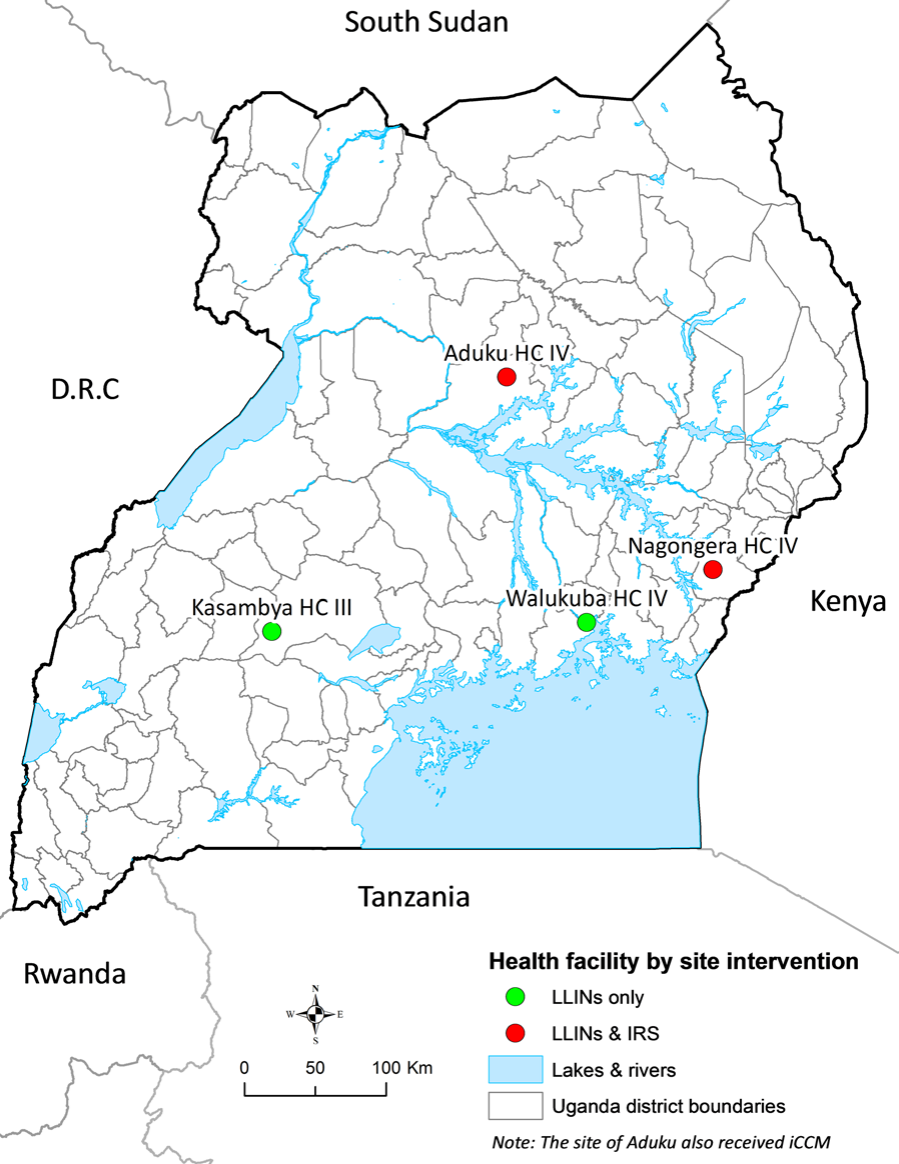
Site locations of the four study health facilities categorized by the main intervention activity used

Four MRCs were selected for this study including: Walukuba in Jinja District, Kasambya in Mubende District, Aduku in Apac District, and Nagongera in Tororo Distict (Fig. 1). These sites were purposively selected because of their temporal representation of the malaria indicators of interest, with data covering the period of interest (January 2009 and July 2018), and have had malaria control intervention activities implemented in each of them. By transmission settings, Walukuba and Kasambya had an estimated annual entomological inoculation rate (aEIR) of less than 10 infective bites per person per year in the early 2000s, while in Aduku and Nagongera aEIR was in excess of 1500 and 550, respectively [31].

### Variable description

Records in the database were classified either as ‘suspected malaria’ or ‘no suspected malaria’. Suspected malaria was defined as patients who: (a) had a laboratory test done for malaria (microscopy or rapid diagnostic test (RDT)); or, (b) were given a clinical diagnosis of malaria in the absence of laboratory testing. All records that did not meet this definition were classified as ‘no suspected malaria’.

Among suspected malaria cases, ‘malaria cases’ were defined as all those patients with positive malaria diagnostic test results (microscopy or RDT). Moreover, ‘malaria negative cases’ were those who were tested for malaria (microscopy or RDT) but had negative test results.

### Description of malaria control interventions at the study sites

**Fig. 2:**
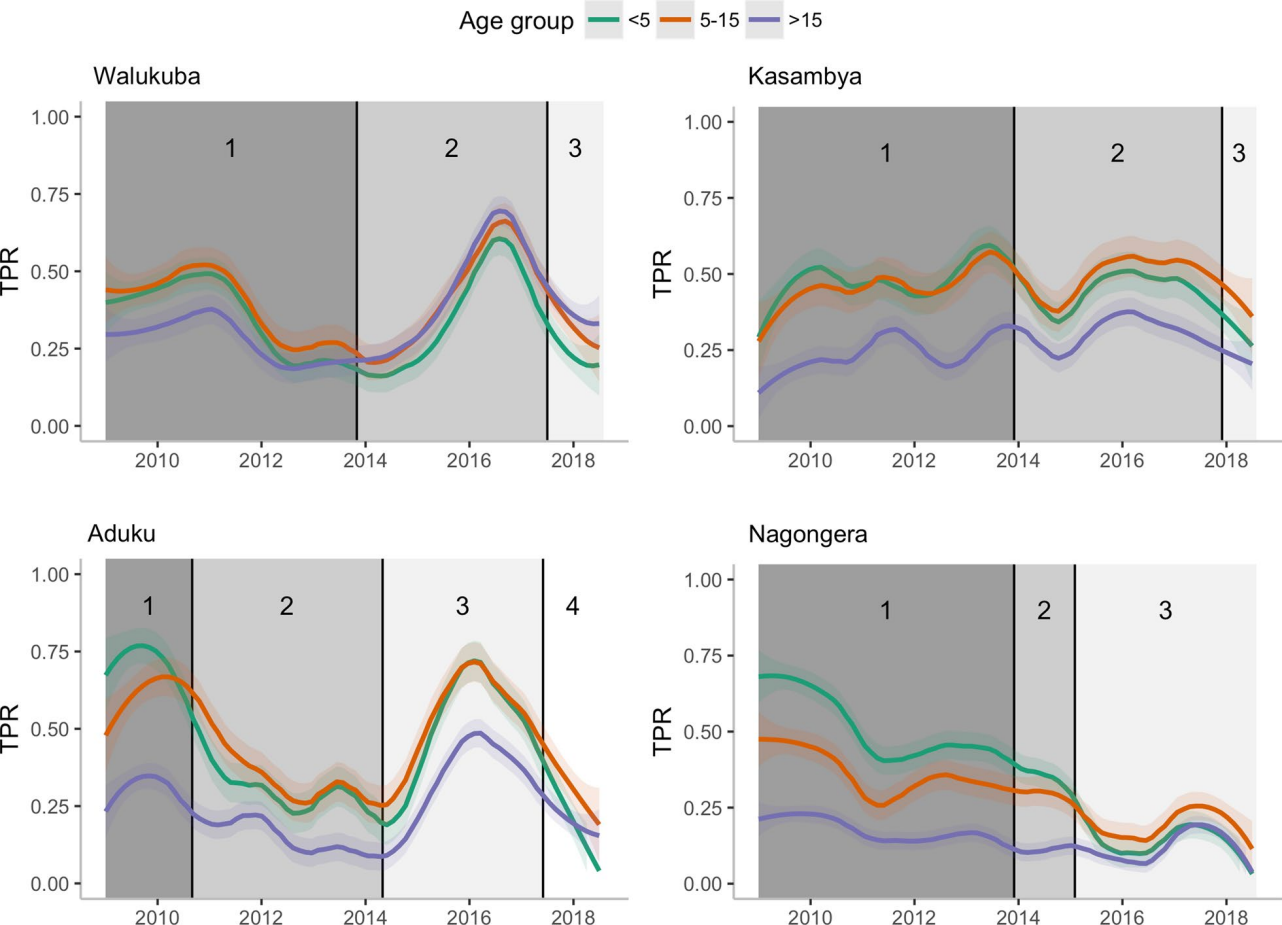
Intervention periods, and TPR trends by age category (< 5, 5–15 and > 15 years) and site. Intervention periods, defined based on malaria control activities per site: Walukuba: LLINs alone. Period 1 (Jan 2009–Oct 2013): Prior to the first mass LLIN distribution campaign conducted during October 2013. Period 2 (Nov 2013–May 2017): Post first mass LLIN distribution and prior to second distribution campaign conducted during May 2017. Period 3 (Jun 2017–Jul 2018): Post second mass LLIN campaign to study endline. Kasambya: LLINs alone. Period 1 (Jan 2009–Nov 2013): Prior to first mass LLIN distribution campaign conducted during November 2013. Period 2 (Dec 2013–Nov 2017): Post first mass LLIN distribution campaign and prior to second distribution campaign conducted during November 2017. Period 3 (Dec 2017–Jul 2018): Post second mass distribution campaign to study endline. Aduku: LLINs, IRS and iCCM. Period 1 (Jan 2009–Aug 2010): One IRS round with the pyrethroid alpha-cypermethrin insecticide was implemented in March 2010. Period 2 (Sep 2010–Apr 2014): Received intense IRS campaign with nine rounds of carbamate bendiocarb approximately 6-monthly, the last of which was conducted during May 2014. The first mass LLIN distribution campaign was also conducted in July 2014. Period 3 (May 2014–May 2017): IRS withdrawal for 3 years till one round of IRS with the organophosphate Actellic insecticide conducted during May 2017. The second mass LLIN distribution campaign was also conducted over this period and iCCM implemented effective 2016. Period 4 (Jun 2017–Jul 2018): Post last round of IRS (May 2017) to study endline. Nagongera: LLINs and IRS. Period 1 (Jan 2009–Nov 2013): Prior to first mass LLIN distribution campaign conducted during November 2013. Period 2 (Dec 2013–Jan 2015): Post first mass distribution and prior to first IRS campaign conducted during January 2015. Period 3 (Feb 2015–Jul 2018): Period where six rounds of IRS were conducted, three rounds with bendiocarb approximately 6-monthly followed by three rounds with Actellic approximately 12-monthly, as well as the second mass LLIN distribution campaign conducted in May 2017

Using available information from the NMCD on when specific interventions were implemented and/or interrupted at each study site, calendar time was divided into three to four different intervention periods per site (Fig. 2). The first period for each site, before large-scale interventions were implemented, is referred to as baseline. During baseline periods, control activities were largely limited to targeted distribution of insecticide-treated nets (ITNs) to vulnerable populations such as children under 5 years of age and pregnant mothers [32, 33]. The subsequent intervention periods were then used to quantify the impact of interventions on the age-distribution of test-confirmed malaria cases.

The main control interventions implemented in Walukuba and Kasambya were two mass LLIN distribution campaigns conducted in 2013 and 2017 at both sites. In addition to LLINs in the same time periods, Aduku and Nagongera also received IRS. The baseline period in Aduku included one round of IRS with the pyrethroid alphacypermethrin insecticide, which was not considered to be effective due to insecticide resistance [30]. This period in Aduku, was followed by nine rounds of IRS with the carbamate bendiocarb insecticide approximately every 6 months, as well as the first mass LLIN distribution campaign. IRS was stopped for 3 years and then a single round of IRS with the organophosphate Actellic insecticide was conducted, immediately followed by the second mass LLIN distribution campaign. In Nagongera, the baseline period was followed by the first mass LLIN distribution campaign and then a sustained period of IRS including three rounds with the bendiocarb insecticide, approximately every 6 months, followed by three rounds with Actellic approximately every 12 months. A second mass LLIN distribution campaign was also conducted in Nagongera during the sustained period of IRS [34].

### Statistical analysis

Data from patients with age missing (0.3%), age over 70 years (1.2%), and follow-up visits for any previously recorded illness episode (0.7%) were excluded from the analyses. First, data were explored by calculating, for each site and intervention period, the total number of patients seen and the proportion of those that were suspected, tested and classified as confirmed malaria cases. To characterize changes in testing practices, proportion of cases that were tested by microscopy vs RDT were calculated. Test positivity rate (TPR) was defined as the proportion of patients tested for malaria that tested positive, a metric often considered a viable proxy for transmission intensity [35].

Trends in the gender distribution of patients with or without suspected malaria and malaria cases were also explored across intervention periods using cross-tabulation with Chi squared tests.

To characterize the impact of control interventions on the age distribution of malaria cases, first, violin density plots were used to visualize changes over the intervention periods and compare the distributions of patients not suspected of malaria to those with confirmed malaria. Second, scatter plots of age (as a continuous variable) and test positivity, stratified by intervention periods, per site were examined. Third, multinomial logistic regression models were fit, by site. The outcome in these models was ‘the age category of confirmed malaria cases’ (under 5 years, 5–15 years, and over 15 years; three age categories being conveniently defined), while the main predictor was the intervention period. Models were adjusted for diagnostic test used (microscopy vs RDT), as well as patient gender. To validate the potential impact of increasing use of RDTs across sites over time on these findings, the multinomial regression models were fit with an interaction between diagnostic test and time. Using the full models, adjusted proportions of malaria cases in each of the age categories per intervention period were predicted. These marginal predictions were made at the mean values of the variables included in the model and analyses performed in R [36] and STATA 15 (Stata Corporation 2017, College Station, TX, USA).

## Results

### Patient composition and attendance by site

Between January 2009 and July 2018, the four health facilities of Walukuba, Kasambya, Aduku, and Nagongera recorded 896,550 patient visits in their outpatient department clinic registers, with over half of them (53%) suspected to be malaria cases. Walukuba recorded the highest number of both patient attendance (323,856) and suspected malaria cases (130,296), while Kasambya had the lowest attendance at 153,811 (Table 1).

**Table 1:** Malaria-associated demographics of study participants for each site, by intervention period

Site	Intervention period	Total observations^a^	Suspected malaria (%)	Tested for malaria (%)^b^	Microscopy (%)	Positive test result (%)^b^
Walukuba	Jan 2009–Oct 2013	183,327	95,080 (51.9%)	92,784 (97.6%)	92,738 (99.9%)	30,785 (33.2%)
	Nov 2013–May 2017	111,506	27,483 (24.7%)	26,051 (94.8%)	24,360 (93.5%)	9239 (35.5%)
	Jun 2017–Jul 2018	29,023	7733 (26.6%)	7024 (90.8%)	5227 (74.4%)	2037 (29.0%)
Kasambya	Jan 2009–Nov 2013	85,200	65,768 (77.2%)	63,479 (96.5%)	60,070 (94.6%)	24,538 (38.7%)
	Dec 2013–Nov 2017	60,488	41,823 (69.1%)	38,328 (91.6%)	30,199 (78.8%)	15,996 (41.7%)
	Dec 2017–Jul 2018	8123	5272 (64.9%)	5083 (96.4%)	1156 (22.7%)	1529 (30.1%)
Aduku	Jan 2009–Aug 2010	34,596	19,024 (55.0%)	17,877 (94.0%)	17,877 (100%)	9921 (55.5%)
	Sep 2010–Apr 2014	71,329	40,428 (56.7%)	40,277 (99.6%)	39,056 (97.0%)	9825 (24.4%)
	May 2014–May 2017	66,092	34,467 (52.2%)	29,410 (85.3%)	17,466 (59.4%)	15,311 (52.1%)
	Jun 2017–Jul 2018	25,389	9013 (35.5%)	8158 (90.5%)	2280 (28.0%)	2189 (26.8%)
Nagongera	Jan 2009–Nov 2013	124,711	82,762 (66.4%)	76,915 (92.9%)	74,959 (97.5%)	27,007 (35.1%)
	Dec 2013–Jan 2015	24,530	15,893 (64.8%)	15,723 (98.9%)	14,874 (94.6%)	3977 (25.3%)
	Feb 2015–Jul 2018	72,236	27,297 (37.8%)	27,064 (99.2%)	16,213 (59.9%)	4309 (15.9%)

^a^Patients seen at the health facility excluding those with a missing record of age

^b^Testing for malaria includes both microscopy and RDT

The highest annual mean number of patients seen was in Walukuba (33,053) followed by Nagongera (22,701), then Aduku (20,079), and least in Kasambya (15,897). Mean monthly patient attendance per year remained fairly constant at all sites except Walukuba where this value peaked in 2011 at 3400 and steadily declined to 1952 by 2018 (Fig. S1, Additional file 1). Mean monthly attendance of patients not suspected of malaria per year increased slowly over time at all sites with cyclic variations (Fig. S2, Additional file 1). From 2009 to 2018, these increases were significant in Kasambya and Aduku though not in Walukuba or Nagongera by Wilcoxon rank-sum test (Table S1, Additional file 1). Conversely, mean monthly attendance of suspected malaria cases per year followed a general decline over time at all sites with cyclic variations (Fig. S3, Additional file 1). From 2009 to 2018, these declines were significant in Walukuba, Aduku and Nagongera, but not in Kasambya (Table S1, Additional file 1).

The majority of non-suspected and suspected malaria cases were female (67 and 63%, respectively). The difference between gender among confirmed malaria cases was smallest among children under 5 years of age (per cent female: Aduku 49%, Nagongera 49%, Walukuba 54%, and Kasambya 55%) and largest among patients over 15 years of age (per cent female: Aduku 79%, Nagongera 75%, Walukuba 63%, and Kasambya 66%). No observable trend in gender overall was found across intervention periods.

### Trends in diagnostic testing over time

Throughout the study period, the majority of laboratory testing for malaria was by microscopy (89%) and the highest and lowest overall testing rates (percentage of suspected malaria cases that received a diagnostic test for malaria) were observed in Walukuba (97%) and Aduku (93%), respectively. Across intervention periods however, the proportion tested by RDT increased at all four sites mostly in the last 4 years of study duration. By the last period, 77% of cases were tested by RDT in Kasambya, 72% in Aduku, 40% in Nagongera, and 25% in Walukuba (Fig. S4, Additional file 1).

### Test positivity rates

Although Aduku and Nagongera were historically the highest transmission settings, the highest TPR was observed in Aduku followed by Kasambya, Nagongera and Walukuba (Table 1). When considering only children under 5 years of age, however, baseline TPR levels were reflective of the historical transmission intensities. Baseline TPR in this group was highest at Nagongera (64%) and Aduku (63%), and lower in Kasambya (37%) and Walukuba (31%).

In all four sites, control interventions were associated with moderate reduction in overall TPR with acyclic secular trends in between. Larger reductions were observed in the two sites where both LLINs and IRS were implemented (Fig. 2). In these sites, the declining trend in TPR is consistent except in Aduku, during the 3 years of withdrawn IRS, characterized by a sharp increase. Between baseline and the last intervention periods, TPR declined in Aduku from 56 to 27% and in Nagongera from 35 to 16%. In the two sites that received LLINs only, a similarly (with acyclic secular but less notably) reducing trend was observed. Between baseline and the last intervention periods, overall TPR decreased in Walukuba from 33 to 29% and in Kasambya from 39 to 30% (Table 1).

Over time and in all four sites, test positivity was seen to decline among the younger children while increasing among older participants. In all sites, a shift in the peak age of test positivity from the youngest to the older ages, was observed between baseline and last intervention period (Figs. S5 and S6, Additional file 1). Interestingly, this pattern was reversed in Aduku during the 3 years when IRS was withdrawn, further confirming the effect of control interventions on test positivity with age (Fig. S6, Additional file 1).

### Differences in age distribution of malaria cases between sites at baseline

**Fig. 3:**
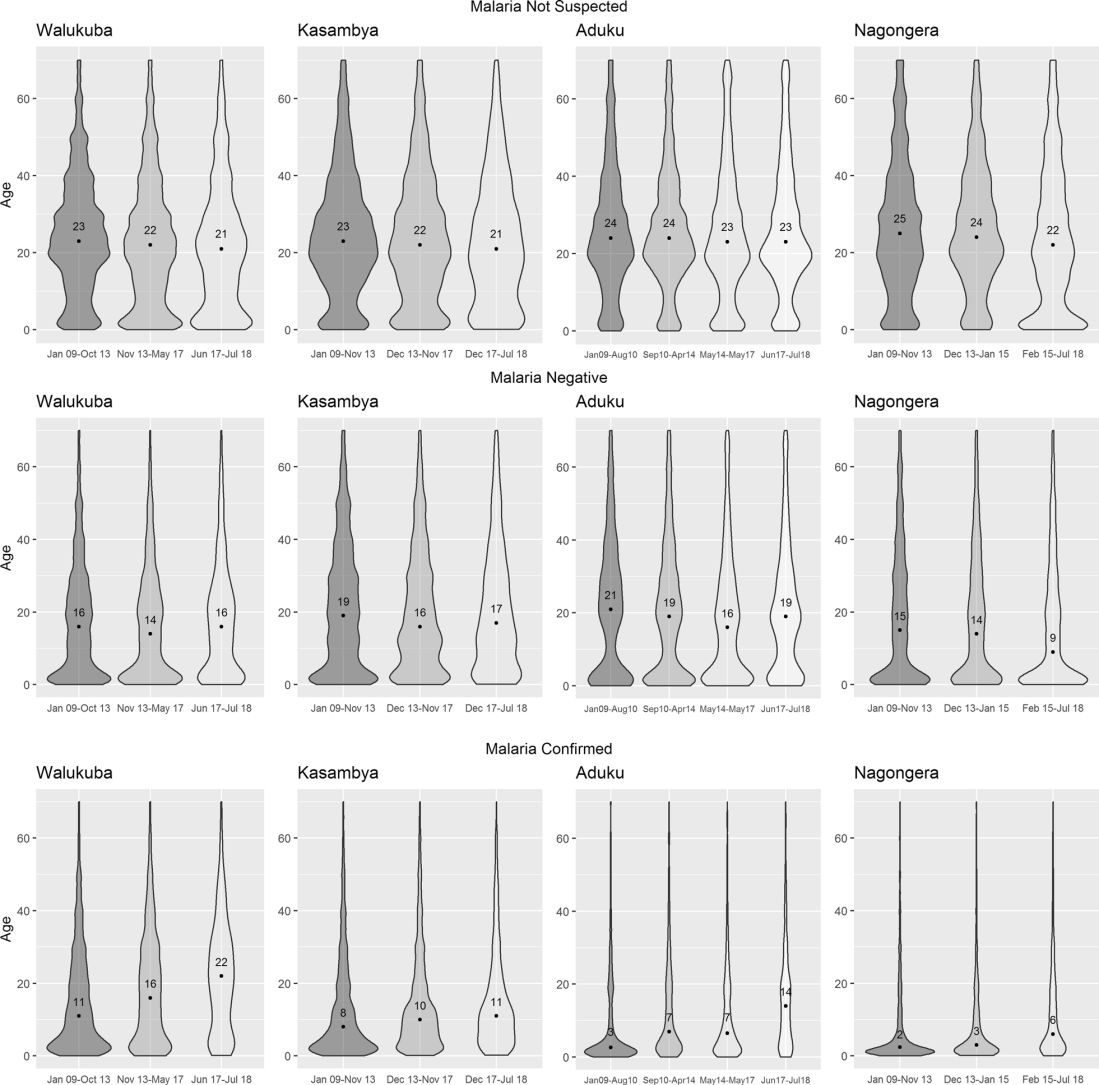
Categorized age distribution across intervention periods, by patient status (not suspected, malaria negative *vs* confirmed)

Although the duration of baseline periods varied between the sites due to the different timing of intervention activities, the age distribution of patients not suspected of malaria was very similar between all four sites at baseline, with median ages ranging from 23 to 25 years (Fig. 3). In contrast, the age distributions of malaria cases varied significantly between sites. These distributions were similar between the highest transmission sites of Nagongera and Aduku, with median ages of 2 and 3 years, respectively. The distributions were also similar, but higher, between the lower transmission sites, with median ages of 8 and 11 years for Kasambya and Walukuba, respectively.

**Fig. 4:**
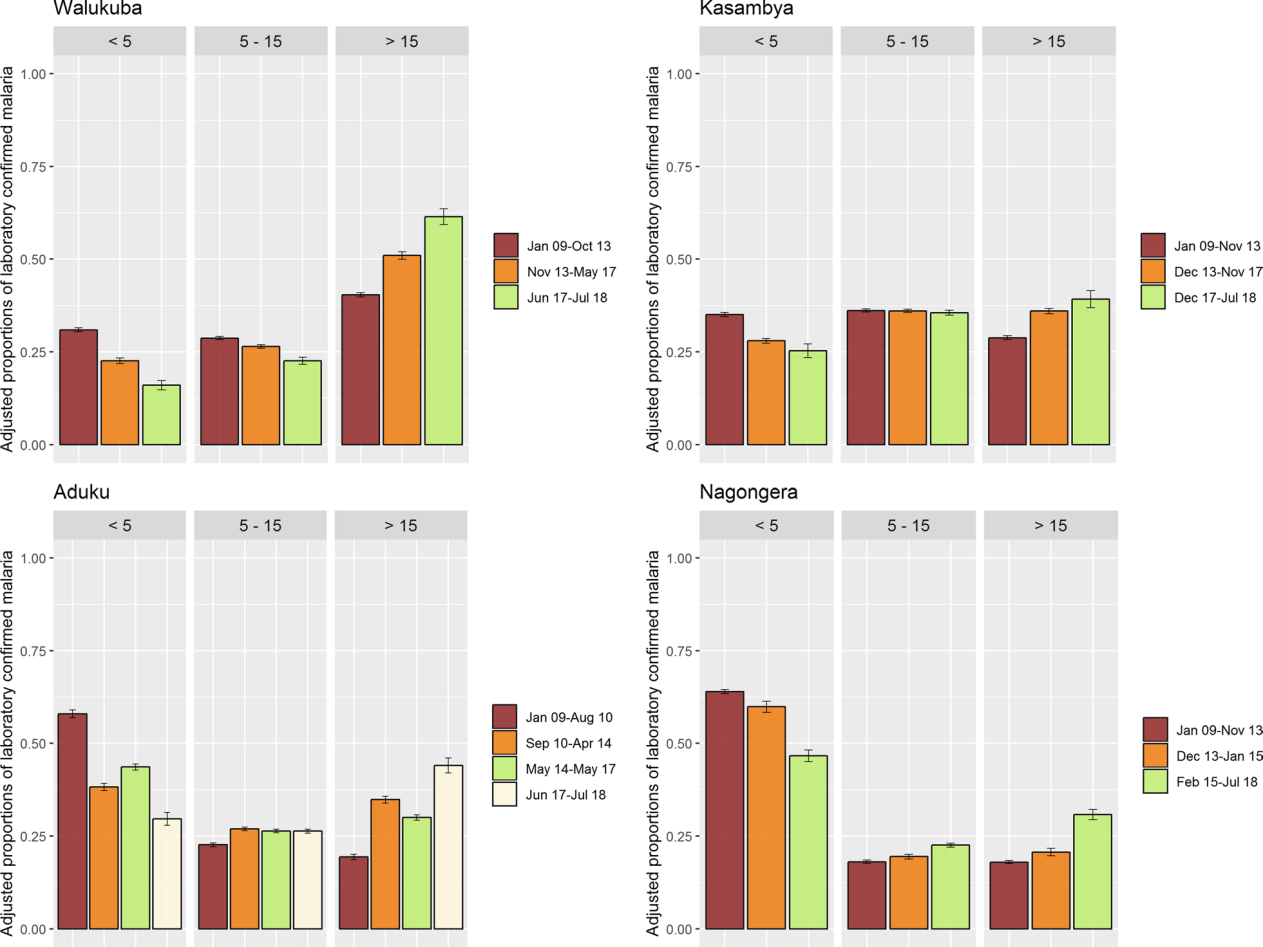
Adjusted marginal probability of confirmed malaria, by intervention period, age and site

Consistent with unadjusted analyses, results from the final adjusted multinomial regression models (adjusting for diagnostic test and gender of patient; evaluated using Akaike’s information criteria and likelihood ratio tests (Table S2, Additional file 1) for model selection; and, using scatter plots with fitted lines for goodness of fit (Fig. S7, Additional file 1) showed that the proportion of malaria cases per age-group were significantly different between sites at baseline. The majority of malaria cases were among children under 5 years of age in the highest transmission sites (Aduku 58% and Nagongera 64%), while the highest proportion of malaria cases was among patients 5–15 years of age in Kasambya (35%), and over 15 years of age in Walukuba (31%) (Fig. 4).

### Changes in age distribution of non-suspected, test-negative, and laboratory confirmed malaria cases over time

The age of patients not suspected of malaria decreased slightly over the study duration at all four sites. Moreover, for malaria negative patients, the age distribution slightly shifted downwards and then upwards at all sites except Nagongera, where it shifted downwards across the intervention periods. In contrast, the age distribution of patients with laboratory confirmed malaria shifted upwards over time at all four sites. Comparing the last observation period to baseline, the median age of patients with malaria increased from 8 (IQR: 2.5–19) to 11 (IQR: 5–21) in Kasambya; 11 (IQR: 3.5–24) to 22 (IQR: 8–32) in Walukuba; 2 (IQR: 1.1–10) to 6 (IQR: 2–18) in Nagongera; and 3 (IQR: 1.2–13) to 14 (IQR: 5–22) in Aduku (Fig. 3).

Across all sites, a progressive decline in the proportion of malaria cases from the youngest age group and a progressive increase in the proportion of cases from the oldest age group were observed. Comparing the last intervention period to baseline, the adjusted proportion of malaria cases among children under 5 years of age decreased from 58% (95% CI 57–59%) to 30% (95% CI 28–31%) in Aduku; 31% (95% CI 30–31%) to 16% (95% CI 15–17%) in Walukuba; 64% (95% CI 63–65%) to 47% (95% CI 45–48%) in Nagongera; and 35% (95% CI 34–36%) to 25% (95% CI 23–27%) in Kasambya. Comparing the same periods, the proportion of malaria cases among patients over 15 years of age increased from 19% (95% CI 19–20%) to 44% (95% CI 42–46%) in Aduku; 40% (95% CI 40–41%) to 61% (95% CI 59–64%) in Walukuba; 18% (95% CI 17–18%) to 31% (95% CI 29–32%) in Nagongera; and, 29% (95% CI 28–29%) to 39% (95% CI 37–41%) in Kasambya.

The upward shift in the age distribution of malaria cases occurred gradually throughout the study periods in all sites except Aduku, where IRS was withdrawn in 2014 for three years (defining the 3^rd^ intervention period) before another round was implemented in 2017. In Aduku, during the intervals from the 2nd to the 3rd intervention periods, the proportion of malaria cases among children under 5 years of age increased from 38% (95% CI 37–39%) to 44% (95% CI 43–44%), followed by a decrease to 30% (95% CI 28–31%) following the last round of IRS. At this site, the proportion of malaria cases among patients over 15 years decreased from 35% (95% CI 34–36%) during the 2nd to 30% (95% CI 29–31%) during the 3rd intervention period, before increasing to 44% (95% CI 42–46%) in the last period (Fig. 4).

The upward shift in age distribution of confirmed malaria occurred consistently in both males and females (Fig. S8, Additional file 1). Whilst the majority of all patients, non-suspected and suspected malaria cases were female across the study durations, small differences were observed in age distribution between males and females that were not suspected to be malaria cases (Fig. S9, Additional file 1), but the age distribution of females was older than that of males among malaria-negative patients across all sites (Fig. S10, Additional file 1). Moreover, models allowing an interaction between gender and intervention period suggest greater increase in proportion of males than females among confirmed malaria cases over time (Fig. S11 and Table S3, Additional file 1).

Overall, Aduku experienced the largest change in the age distribution of malaria cases throughout the study period. The odds of an upward shift in the age category of confirmed malaria cases in the last relative to the baseline intervention periods were 3.27 (95% CI 2.97–3.61) in Aduku, 2.35 (95% CI 2.14–2.58) in Walukuba, 2.03 (95% CI 1.90–2.17) in Nagongera, and 1.59 (95% CI 1.44–1.76) in Kasambya (Table 2). Whereas the interaction between intervention time and diagnostic test used was significant in Kasambya and Nagongera, the same did not notably impact the overall effect observed (Table S4, Additional file 1). Also, RDT use increased gradually at all sites, reaching 20% only in the last three to four years of the study except Aduku (Fig. S4, Additional file 1).

**Table 2:** Association between being malaria confirmed within ages (< 5, 5–15, > 15 years) and control intervention period

Factor	Categories	Odds	95% CI	P value
Walukuba				
Gender	Male	1	Ref	
	Female	1.34	1.29–1.39	< 0.001
Malaria test done	Microscopy	1	Ref	
	RDT	2.11	0.61–7.26	0.237
Intervention period	Baseline	1	Ref	
	First period	1.55	1.48–1.62	< 0.001
	Last period	2.27	2.05–2.51	< 0.001
Kasambya				
Gender	Male	1	Ref	
	Female	1.39	1.34–1.45	< 0.001
Malaria test done	Microscopy	1	Ref	
	RDT	0.98	0.93–1.03	0.368
Intervention period	Baseline	1	Ref	
	First period	1.39	1.34–1.44	< 0.001
	Last period	1.59	1.44–1.76	< 0.001
Aduku				
Gender	Male	1	Ref	
	Female	2.64	2.54–2.75	< 0.001
Malaria test done	Microscopy	1	Ref	
	RDT	1.26	1.19–1.32	< 0.001
Intervention period	Baseline	1	Ref	
	First period	2.23	2.11–2.35	< 0.001
	Second period	1.78	1.68–1.89	< 0.001
	Last period	3.27	2.97–3.61	< 0.001
Nagongera				
Gender	Male	1		
	Female	2.18	2.09–2.28	< 0.001
Malaria test done	Microscopy	1		
	RDT	1.24	1.15–1.34	< 0.001
Intervention period	Baseline	1		
	First period	1.19	1.11–1.27	< 0.001
	Last period	2.03	1.90–2.17	< 0.001

## Discussion

The impact of reductions in malaria transmission following the scale-up of malaria control interventions on the age distribution of malaria cases, was investigated using routine surveillance data from four sentinel health facilities in Uganda. The study included data from sites with historically varied transmission intensity where large-scale programmatic control interventions of either LLINs alone or LLINs plus IRS were implemented over the approximately 10-year study period.

Study findings provide empirical evidence of rapid shifts in the malaria burden to older individuals, following implementation of effective malaria control interventions. Over time, the proportion of test confirmed malaria cases progressively decreased in children under 5 years of age while it progressively increased in those over 15 years of age, irrespective of transmission settings. This is also reflected by subtle but greater decline in TPR among children than adults. The absence of similar changes in age patterns among patients not suspected of malaria suggests that the primary drivers of this shift were declines in malaria transmission intensity associated with effective control interventions and not changes in patient demographics. The reverse shift observed in Aduku during a period of malaria resurgence after three years of interrupted IRS [37] provides further support for this conclusion.

Many factors may have contributed to the shift in age distribution of malaria cases in this study. For many endemic infectious diseases, decreases in transmission are expected to result in increased age of infection and cases [38]. For pathogens like *P. falciparum*, where partial immunity develops gradually as a consequence of repeated exposure, waning immunity due to decreased infection rates may lead to reduced ability to control parasites [39]. This waning immunity may result in a relative increase in the disease burden among adolescents and adults [12, 40]. Concentration of the malaria burden among older age groups following reduced transmission has been predicted in other studies [8], and seen in children for both severe and non-severe outcomes [10, 41].

Behavioural factors may have also contributed to rapid shifts in the age distribution of malaria. A reduced proportion of cases among children under 5 years of age may have been due to an increased use of LLINs among this age group relative to older age groups [22, 41, 42]. In adults, behavioural factors including travel, leisure and social activities, and occupational activities such as agriculture or night-time work may have increased the risk of exposure outside the household as compared to children. Travel has been reported as a risk factor for *P. falciparum* infection in East and Southern Africa [43, 44] and for cases of imported malaria being older than those not imported in Southern Africa [45]. Whereas occupational hazards were not evaluated in this study, considerable occupational risk of malaria has been documented among mobile male workers in Asia [46, 47] and populations involved in agriculture in Africa [48, 49].

Results suggest that implementation of LLINs plus IRS was associated with larger decreases in transmission and larger shifts in age distribution of cases than LLINs alone. However, among LLIN-only sites, Walukuba recorded a much larger shift than Kasambya and in Walukuba the magnitude of the shift was comparable to that of Nagongera, a site with both LLINs and IRS. This suggests that other non-intervention factors for example urbanization [50] may have contributed to the shifts, consistent with other reported findings [38, 51].

Findings from this study showed that a significantly higher proportion of females, as compared to males, seem to seek care for febrile illnesses among the school aged (5–15 years) and adults (over 15 years old) at all sites. This is consistent with the generally older age distribution of females among both the confirmed and the negative malaria cases, but similar age distribution of males and females among those not suspected of malaria. However, shifts in the age distribution of malaria cases observed after control interventions seem to have disproportionately affected males, suggesting a role of gender-based occupational or behavioural differences. Nevertheless, reported greater involvement of females in agriculture in the region [52] than males, as well as documented associations between occupation and education status of mothers and malaria infection risk of families [53] may explain the prevailing significantly high proportion of older females and hence the need for continued interventions that address this vulnerable group.

This study had limitations. First, without well-defined catchment populations for the study sites, it was not possible to estimate changes in malaria incidence over time or effects of environmental factors within the catchment areas and limiting the study to describing temporal shifts in the age distribution of laboratory confirmed cases of malaria. Additional research is necessary to determine impacts of reduced transmission on incidence of malaria. Second, the increased use of RDTs over time, may have influenced results from this study through varied sensitivity [54]. Nevertheless, in regression analyses, diagnostic test used was accounted for. Moreover, patterns seen among suspected and confirmed cases were absent in the cases not suspected of malaria, suggesting that the impact of diagnostic method would be minimal. Third, inability to account for levels of uptake of the interventions implemented at the four study sites could have masked some important variations. However, the community-wide benefit of reduced vector populations [55], coupled with the multiple rounds of interventions, was believed to have mitigated this effect and thus its impact on these results. Fourth, whereas fever or history of fever in the past 48 h is the main indicator of suspected malaria, this specific measure was not consistently recorded in the health facilities, and so inconsistency in consideration of the diagnosis may have impacted results from this study. Nevertheless, other proxy indicators of suspected malaria were also considered to ensure a fairly complete capture of these cases. Lastly, having analysed health facility surveillance data, this would only include participants that seek care in the public health facilities. As such community level interventions, especially iCCM that target young children could have had an impact on these findings. However, iCCM was limited to one of the four sites and for less than 2 years.

## Conclusions

The findings from this study have important implications regarding targeted malaria control interventions that have historically focused on children under 5 years of age. Whereas these efforts need to continue, new strategies may be necessary to address the shift in burden to older age groups following the implementation of successful malaria control interventions. For instance, extending iCCM for malaria to older age groups after intensive IRS, with the post-IRS duration often associated with malaria resurgence. These findings also have implications for the optimal allocation of health care resources for the diagnosis, treatment and control of malaria amidst changing transmission. Further, they highlight the usefulness of considering age distribution in surveillance and monitoring change, and the role of surveillance in understanding the epidemiology of malaria.

## Supplementary information

**Additional file 1: Figure S1.** Trends in mean monthly overall patient attendance per year, stratified by site. **Figure S2.** Trends in mean monthly attendance of patients not suspected of malaria per year, by site. **Figure S3.** Trends in mean monthly suspected malaria patients per year, stratified by site. **Table S1.** Changes in attendance of patients suspected versus not suspected of malaria over-time, comparing mean monthly attendance between first and last calendar years of study duration. **Figure S4.** Age distribution of test confirmed malaria cases, by sex and site. **Figure S5.** Age distribution of patients not suspected of malaria by sex and site. **Figure S6.** Age distribution of patients that tested negative for malaria, by sex and site. **Figure S7.** Adjusted marginal probability of test confirmed malaria, by sex, intervention period, age, and site. **Table S2.** Multivariable association between age (in three categories) and covariates of interest among malaria confirmed cases, accounting for effect modification of intervention periods on sex. **Table S3.** Association between age (in three categories) and covariates of interest among malaria confirmed cases, fitting an interaction between diagnostic test used (B/S vs. RDT) and intervention duration. **Figure S8.** Trends in the annual proportion of RDT use among tested participants, stratified by site. **Figure S9.** Scatter plot of age with test positivity for LLINs only sites, stratified by intervention period. **Figure S10.** Scatter plot of age with test positivity for (LLIN plus IRS) sites, by intervention period.

## Data Availability

The datasets used and/or analyzed for this study are available from the corresponding author on reasonable request.

## Abbreviations

ACT: Artemisinin-based combination therapy
aEIR: Annual entomological inoculation rate
CI: Confidence interval
HMIS: Health Management Information System
iCCM: Integrated Community Case Management
IQR: Inter quartile range
IRS: Indoor residual spraying of insecticide
ITN: Insecticide treated net
LLIN: Long-lasting insecticidal net
MIS: Malaria indicator survey
MRC: Malaria Reference Centre
NMCD: National Malaria Control Division
OPD: Outpatients department
RDT: Rapid diagnostic test
TPR: Test positivity rate
UMSP: Uganda Malaria Surveillance Project
WHO: World Health Organization
