# Supplementary material for: Rapid shifts in the age-specific burden of malaria following successful control interventions in four regions of Uganda
Source: Malar J. 2020 Mar 30;19:128. doi: 10.1186/s12936-020-03196-7 (PMC7106889; doi:10.1186/s12936-020-03196-7)
Supplement: Supplementary file 1 — Additional file 1: Figure S1. Trends in mean monthly overall patient attendance per year, stratified by site. Figure S2. Trends in mean monthly attendance of patients not suspected of malaria per year, by site. Figure S3. Trends in mean monthly suspected malaria patients per year, stratified by site. Table S1. Changes in attendance of patients suspected versus not suspected of malaria over-time, comparing mean monthly attendance between first and last calendar years of study duration. Figure S4. Age distribution of test confirmed malaria cases, by sex and site. Figure S5. Age distribution of patients not suspected of malaria by sex and site. Figure S6. Age distribution of patients that tested negative for malaria, by sex and site. Figure S7. Adjusted marginal probability of test confirmed malaria, by sex, intervention period, age, and site. Table S2. Multivariable association between age (in three categories) and covariates of interest among malaria confirmed cases, accounting for effect modification of intervention periods on sex. Table S3. Association between age (in three categories) and covariates of interest among malaria confirmed cases, fitting an interaction between diagnostic test used (B/S vs. RDT) and intervention duration. Figure S8. Trends in the annual proportion of RDT use among tested participants, stratified by site. Figure S9. Scatter plot of age with test positivity for LLINs only sites, stratified by intervention period. Figure S10. Scatter plot of age with test positivity for (LLIN plus IRS) sites, by intervention period. [file 12936_2020_3196_MOESM1_ESM.doc]

**Consideration of trends in attendance**

Figure S1. Trends in mean monthly overall patient attendance per year, stratified by site.


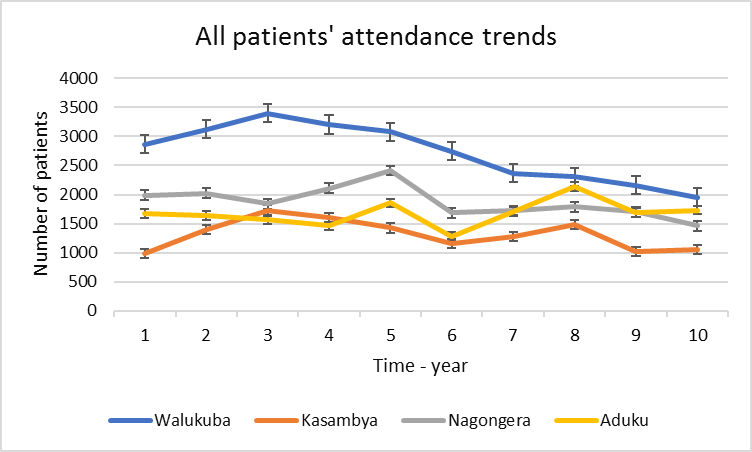


The years on the x-axes in Figures S1 to S4 are represented as 1 to 10 corresponding to the years 2009 to 2018 while the number of patients and/or cases on the y-axis represent monthly average number per year in the study duration.

Figure S2. Trends in mean monthly attendance of patients not suspected of malaria per year, by site


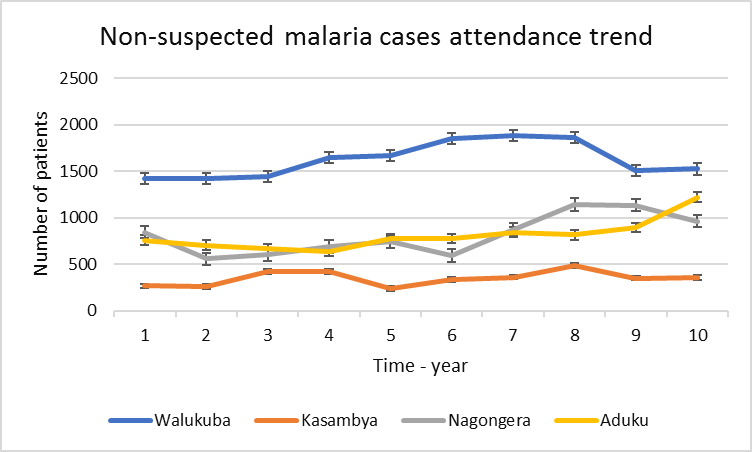


Figure S3. Trends in mean monthly suspected malaria patients per year, stratified by site


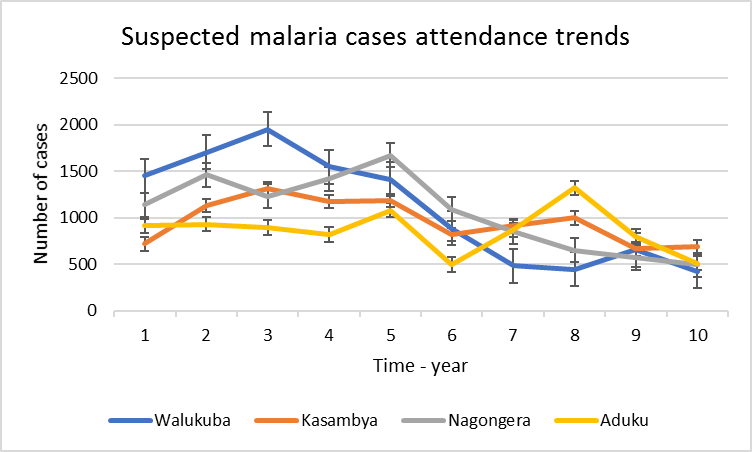


Table S1. Changes in attendance of patients suspected versus not suspected of malaria over-time, comparing mean monthly attendance between first and last calendar years of study duration.

| **Site** | **Patient category** | **Mean monthly attendance per year (SD)** | | **Wilcoxon rank-sum test** |
| --- | --- | --- | --- | --- |
|  |  | **2009** | **2018** | **P value** |
| Walukuba | Not suspected of malaria | 1418 (178) | 1525 (257) | 0.353 |
|  | Suspected malaria | 1452 (372) | 427 (84) | <0.001 |
| Kasambya | Not suspected of malaria | 268 (73) | 360 (73) | 0.023 |
|  | Suspected malaria | 722 (186) | 692 (280) | 0.866 |
| Aduku | Not suspected of malaria | 761 (175) | 1222 (156) | <0.001 |
|  | Suspected malaria | 915 (286) | 512 (99) | 0.003 |
| Nagongera | Not suspected of malaria | 846 (140) | 965 (134) | 0.108 |
|  | Suspected malaria | 1139 (157) | 496 (183) | <0.001 |

Figure S4. Trends in the annual proportion of RDT use among tested participants, stratified by site.


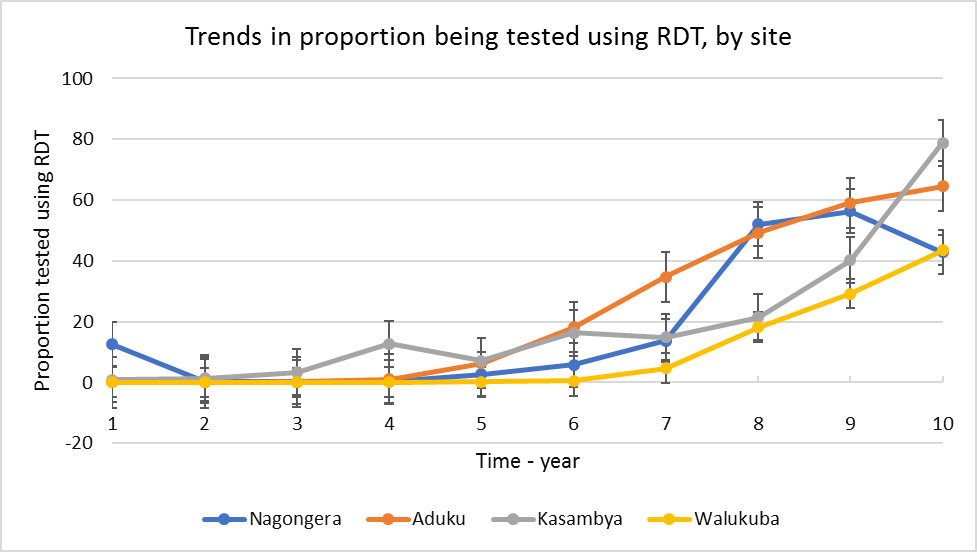


There was little to no RDT use in the first five years of this study duration and most sites did not get to 20% use of RDTs till after 2015 (year number 7 in Figure S4). The predominant diagnostic test used in this study therefore, was microscopy.

Figure S5. Scatter plot of age with test positivity for LLINs only sites, stratified by intervention period.


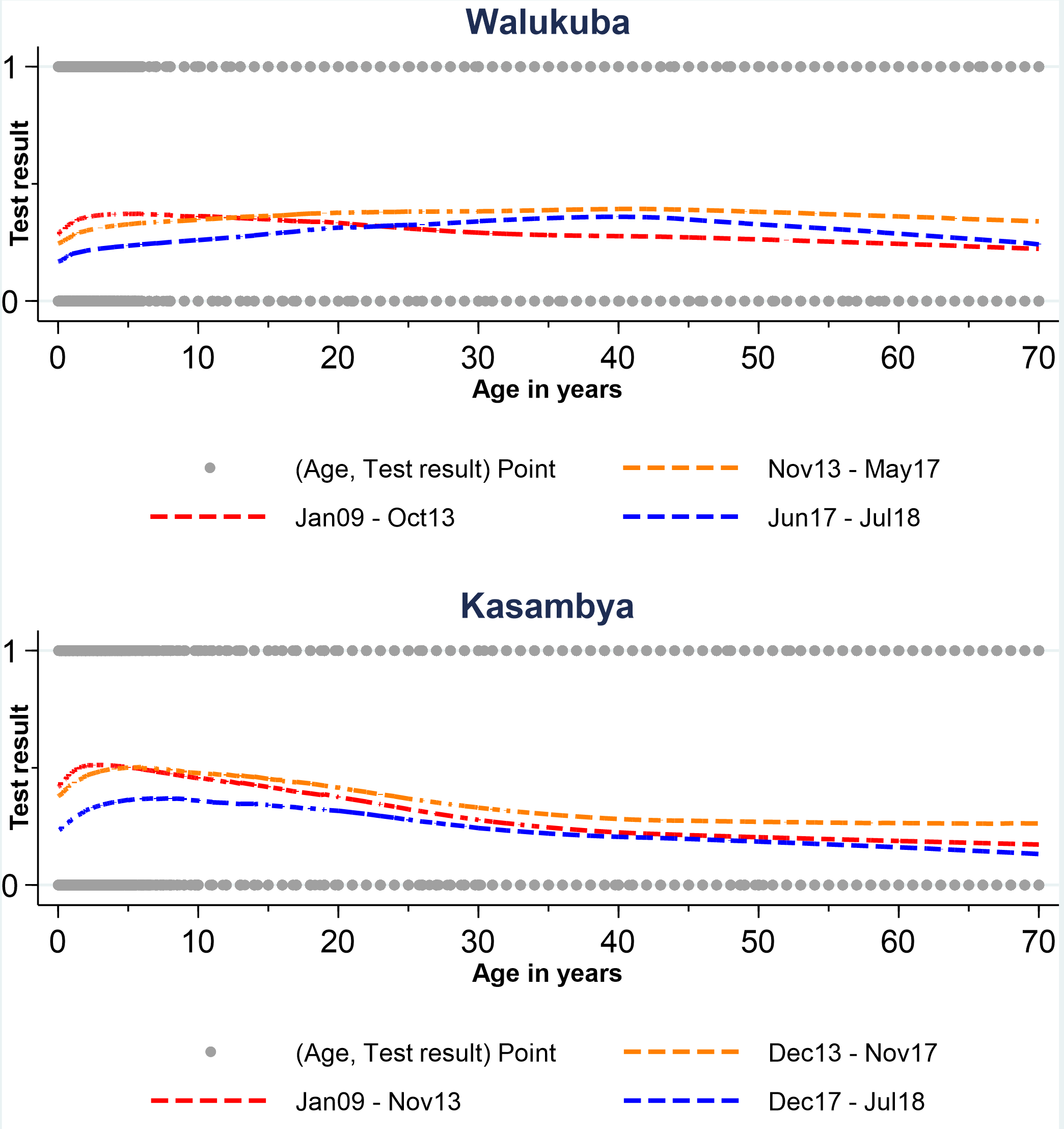


In Figure S5, the x-axis represents the age of the participants (70 years and younger) and the y-axis, the test result from malaria diagnostic tests performed. From these tests, 0 corresponds to a negative result while 1 represents a positive result. The gray points are the (age, test result) coordinates of the scatter plot and the dashed curves the relationship fitted using the Lowess smoother function. The red dashed curve represents the relationship of the baseline period, the orange dashed curve – the first intervention period, and the blue dashed curve – the last intervention period of the study duration. By the last intervention period, positivity among the youngest participants was lower than during baseline and the largest shift was observed in Walukuba where in the last intervention period the peak age of malaria positivity was over 40 years compared to among under 5 years at baseline.

Figure S6. Scatter plot of age with test positivity for (LLIN plus IRS) sites, by intervention period.


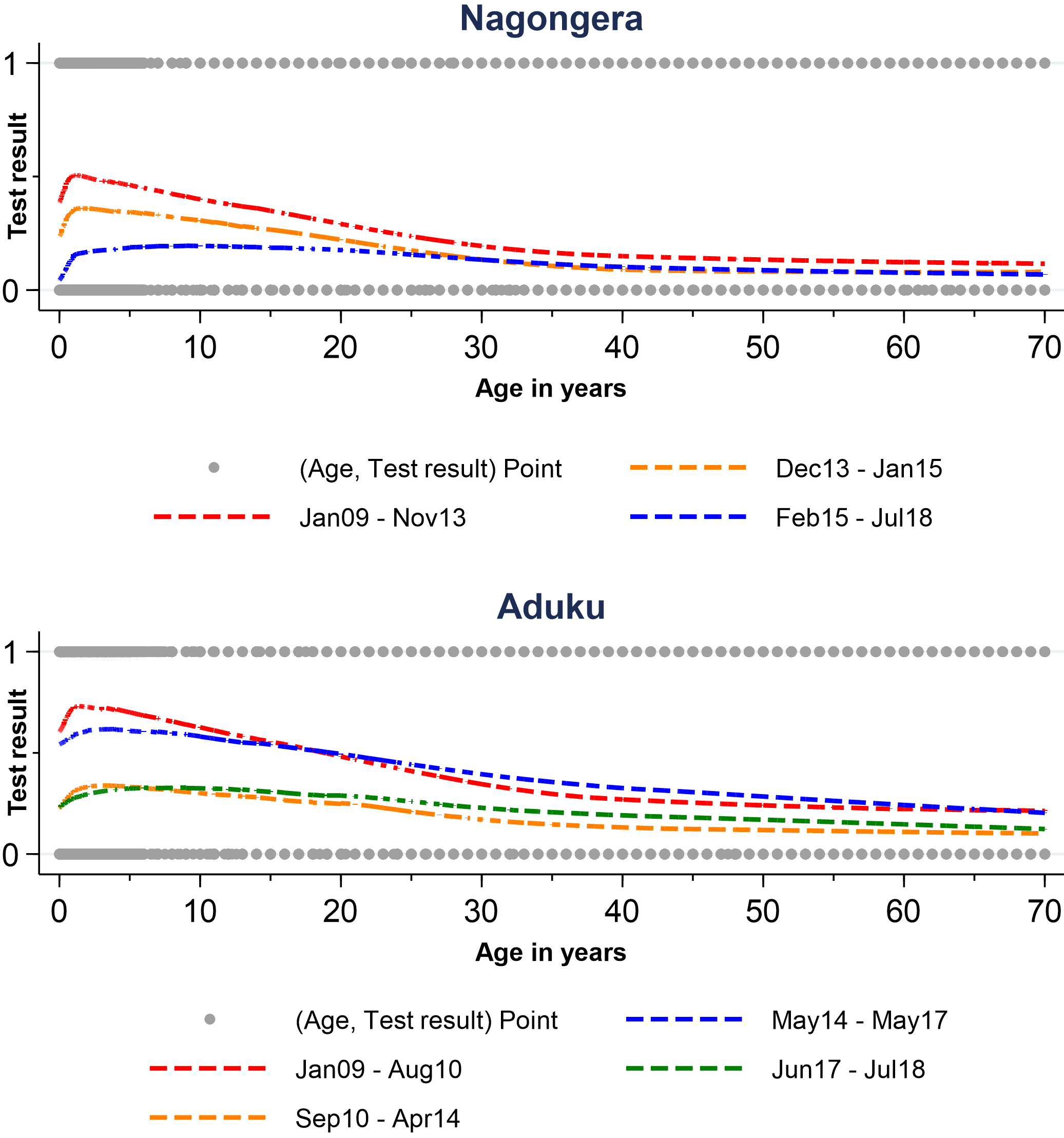


In this case (Figure S6), the x-axis represents the age of participants and the y-axis, the test result from malaria diagnostic test performed. From these tests, 0 on the x-axis corresponds to a negative result while 1 represents a positive result. The gray points are the (age, test result) coordinates of the scatter plot and the dashed curves the relationship fitted using the Lowess smoother function. The red dashed curve represents the relationship for the baseline period, the orange dashed curve – the first intervention period, and the blue dashed curve – the last intervention period in Nagongera, but the second intervention period in Aduku. For Aduku, the green dashed curve represents the last intervention period of the study duration.

For all sites in Figure S6, larger decreases in test positivity among the younger children were observed compared to the sites in Figure S5 above. When IRS was withdrawn in Aduku, however, a pattern similar to that during baseline was observed (represented by the blue dashed curve). During the last intervention period, once IRS was resumed and partly supplemented by integrated community case management iCCM for malaria, the pattern (represented by the green dashed curve) was comparable to the first intervention period when intense IRS was implemented (represented by the orange dashed curve).

**Model evaluation for the adjusted multinomial regression**

Table S2. Model selection for the final model based on performance with inclusion of the main exposure metric of the intervention over time.

| **Site** | **Model** | **AIC** | **Model 1 is nested in the final model** |
| --- | --- | --- | --- |
| Walukuba | Model 1 | 89998.26 | Chi-sq. = 582.45; P<0.001 |
|  | Model 2 (final) | 89419.82 |  |
| Kasambya | Model 1 | 91870.95 | Chi-sq. = 323.16; P<0.001 |
|  | Model 2 (final) | 91551.79 |  |
| Aduku | Model 1 | 76239.23 | Chi-sq. = 1051.01; P<0.001 |
|  | Model 2 (final) | 75194.22 |  |
| Nagongera | Model 1 | 64666.48 | Chi-sq. = 417.42; P<0.001 |
|  | Model 2 (final) | 64253.06 |  |

Model 1 = The model adjusted for gender (male vs. female) and diagnostic test used (microscopy vs. RDT) only

Model 2 = Final model that was adjusted for gender and diagnostic test used, as well as intervention period. This model was found to improve model 1 and therefore the final one based on both AIC and likelihood ratio test evaluations.

Figure S7. Evaluation of model goodness of fit by examining relationship between model predicted proportions of confirmed malaria cases by age category (<5, 5-15, & >15years) adjusted for gender and diagnostic test used, and crude proportion of confirmed malaria cases across intervention periods, by site.

| 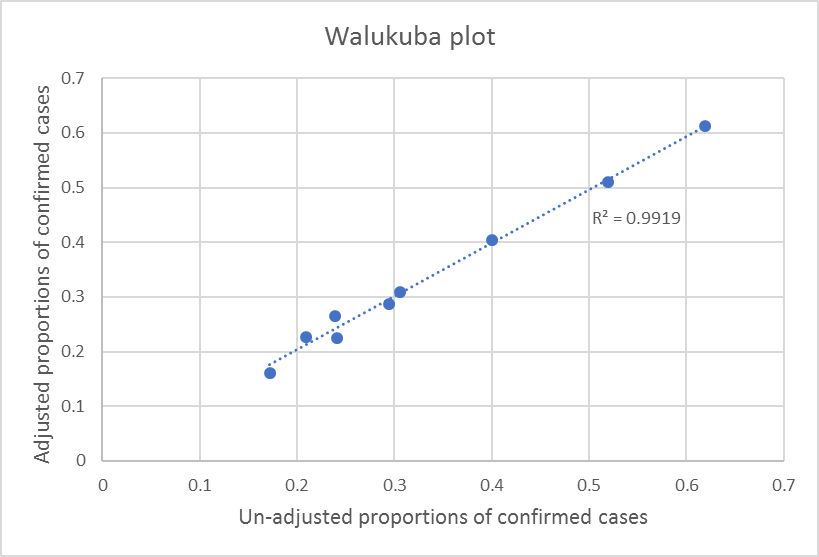 | 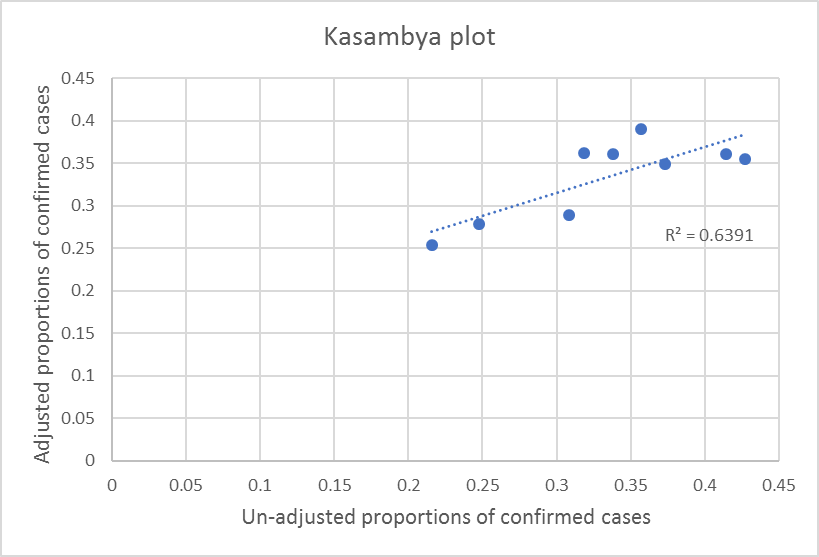 |
| --- | --- |
| 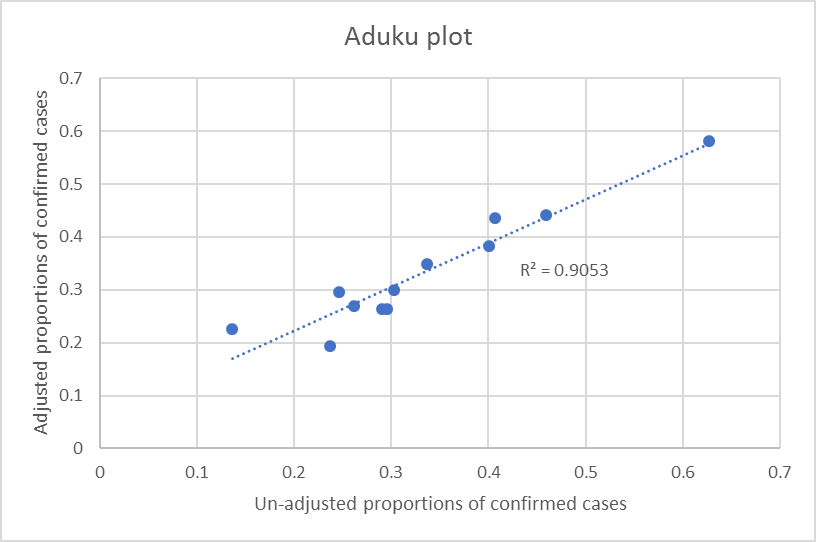 | 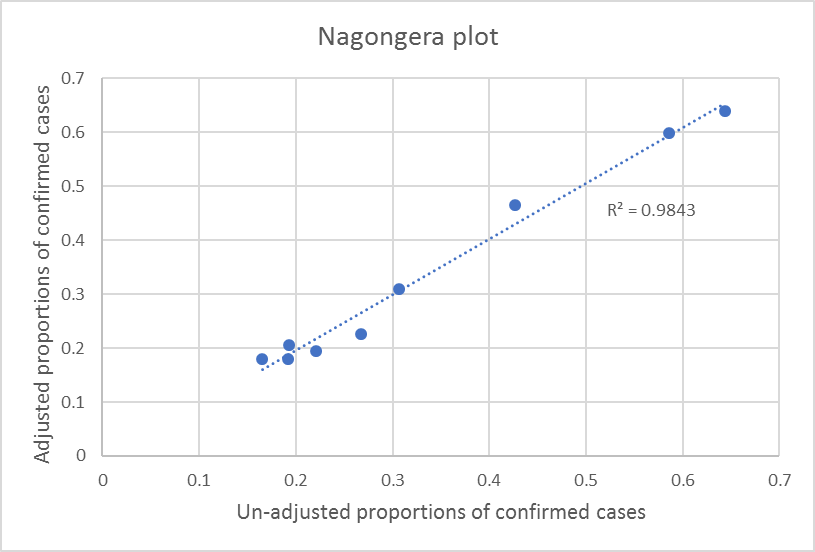 |

At all sites, multinomial models are seen to fit the data very well, best in Walukuba, Nagongera and Aduku and a little less so in Kasambya.

**Consideration of age distribution by gender of patients.**

Figure S8. Age distribution of test confirmed malaria cases, by gender and site across intervention periods.


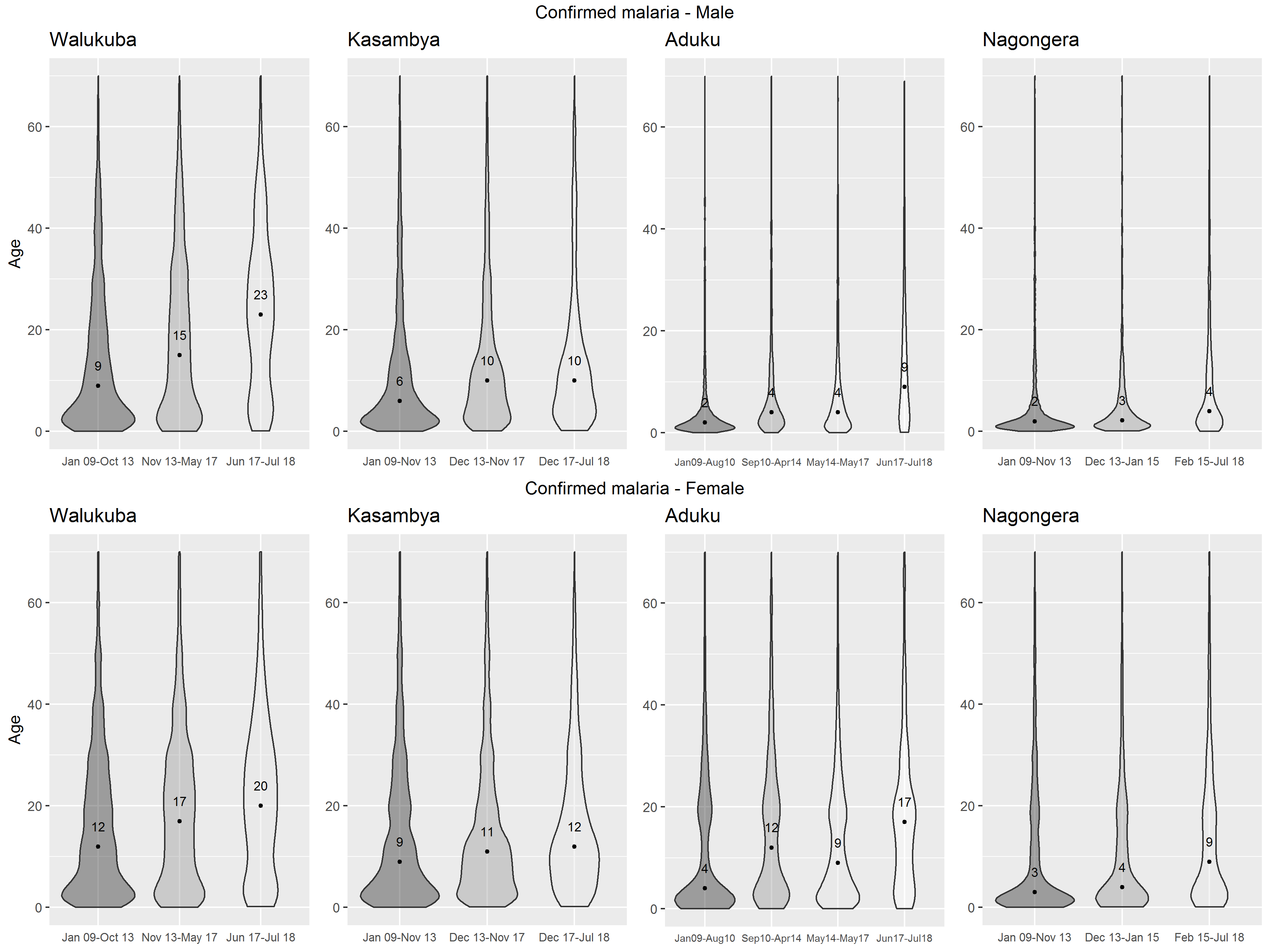


Figure S9. Age distribution of patients not suspected for malaria, by gender and site across intervention periods.


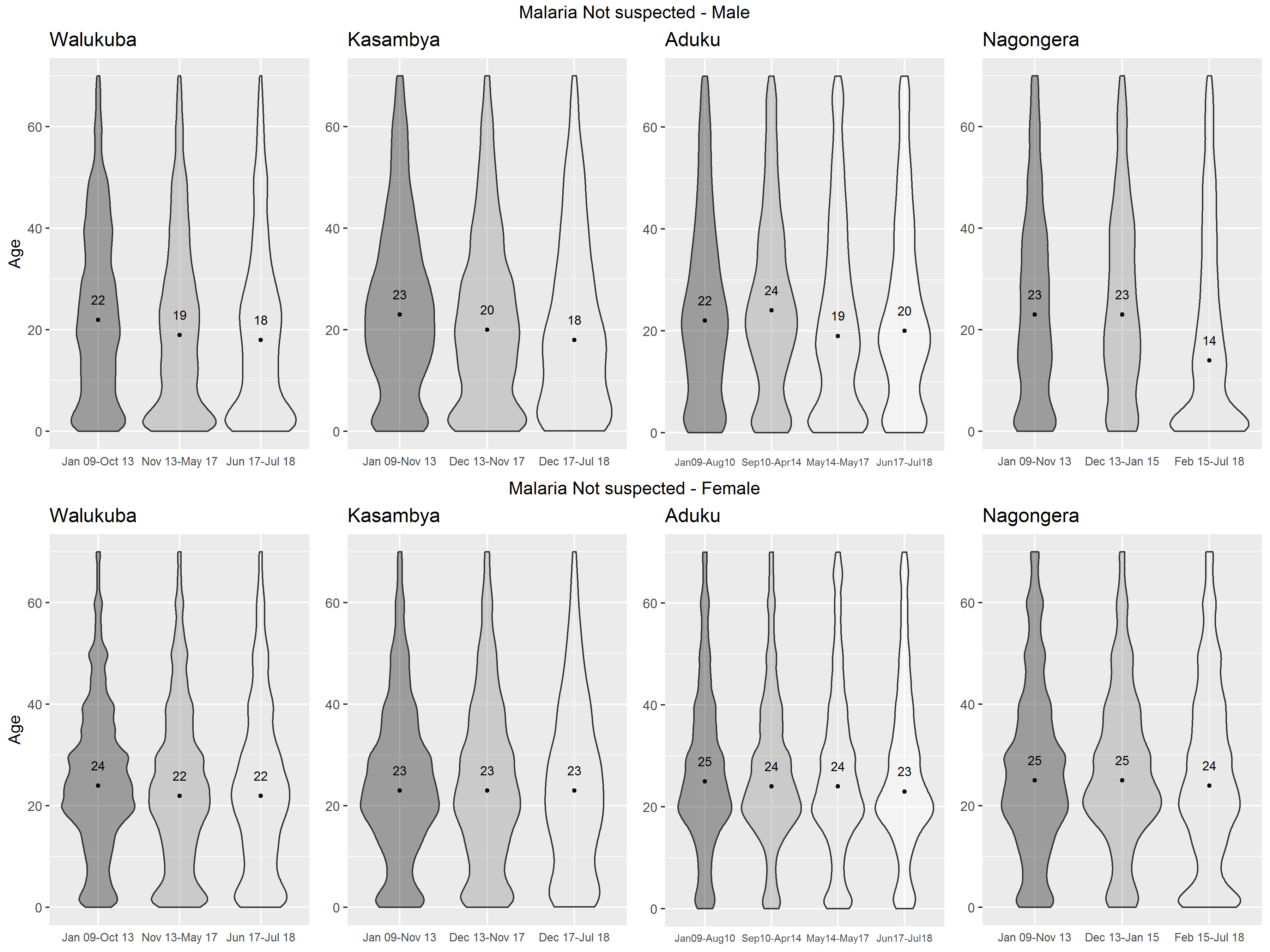


Figure S10. Age distribution of patients that tested negative for malaria, by gender and site across intervention periods.


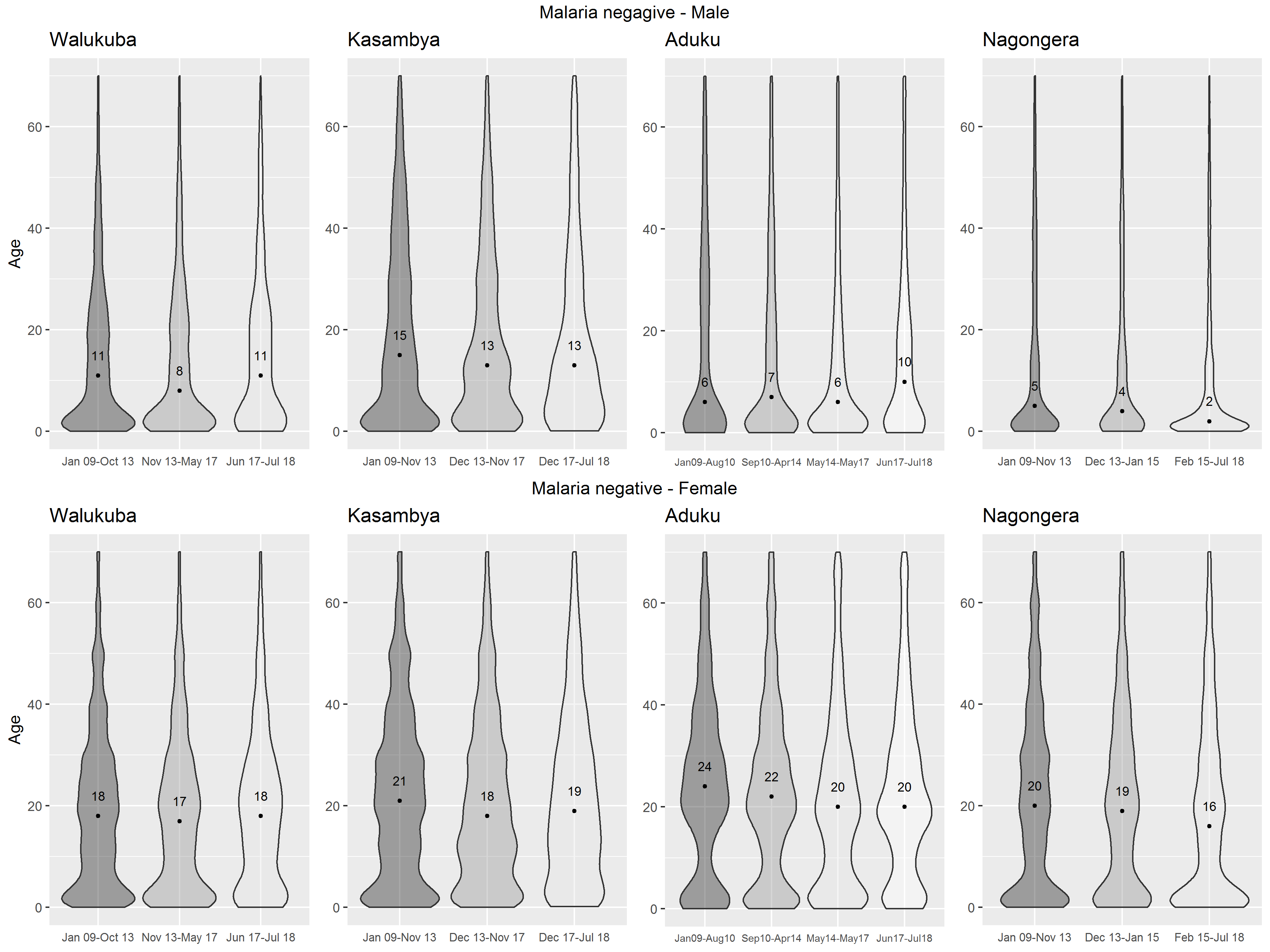


Figure S11. Adjusted marginal probability of test confirmed malaria, by gender, intervention period, age, and site.


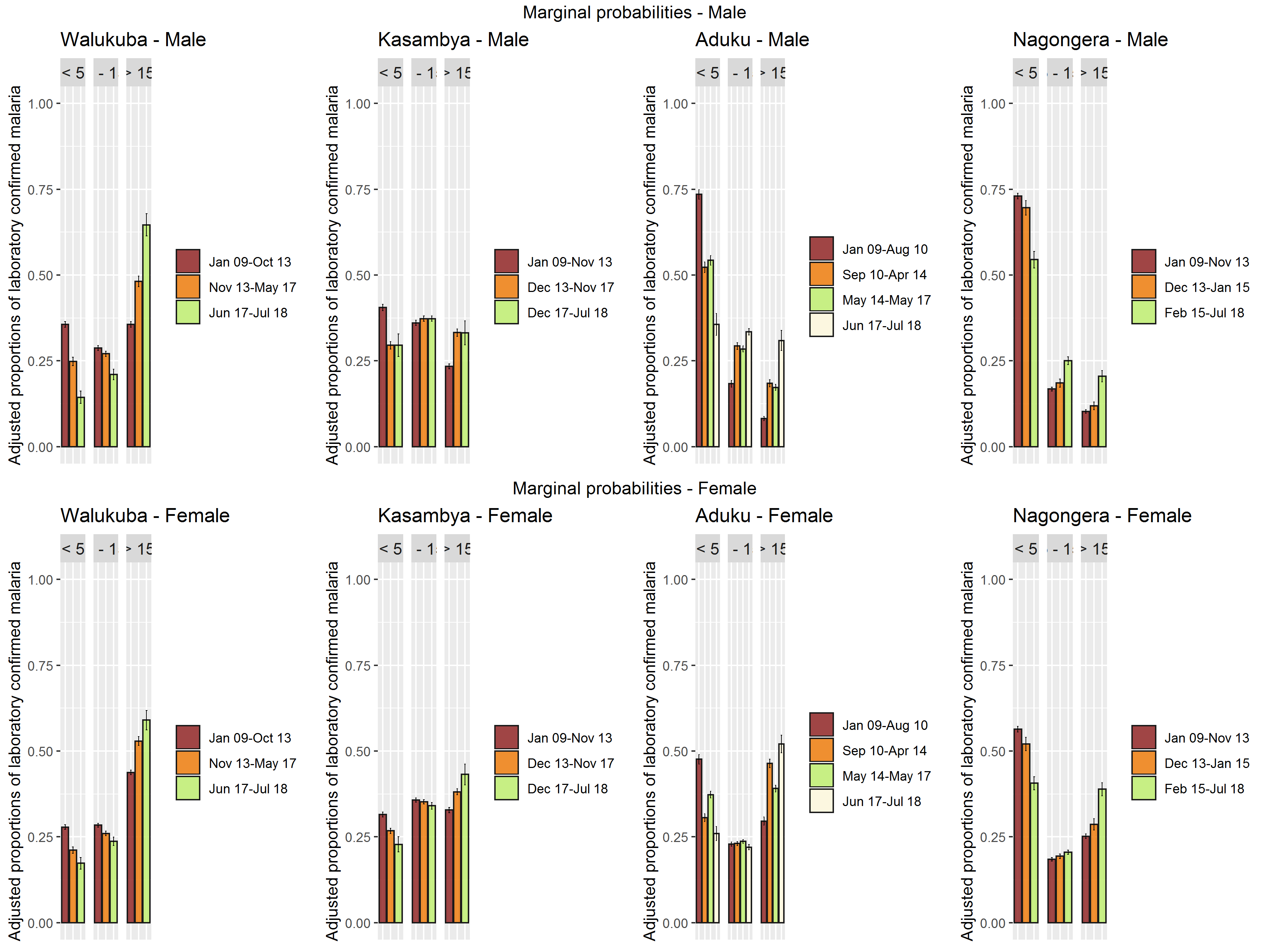


The three age categories include: under 5 years, 5-15 years, and over 15 years in each site while intervention periods are arranged by dates.

Table S3. Multivariable association between age (in three categories) and covariates of interest among malaria confirmed cases, accounting for effect modification of intervention periods on gender.

|  | **Covariate category** | **Multi-variable OR** | **95% CI** | **P - value** |
| --- | --- | --- | --- | --- |
| **Walukuba** | | | | |
| Diagnostic test done | B/S | 1 | *Ref* |  |
|  | RDT | 1.01 | 0.91 - 1.13 | 0.844 |
| Gender | Male | 1 | *Ref* |  |
|  | Female | 1.41 | 1.36 - 1.47 | <0.001 |
| Intervention period | Jan 2009 - Oct 2013 | 1 | *Ref* |  |
|  | Nov 2013 - May 2017 | 1.66 | 1.55 - 1.78 | <0.001 |
|  | Jun 2017 - Jul 2018 | 3.27 | 2.83 - 3.77 | <0.001 |
| Effect of gender by intervention period | (Jan 2009 - Oct 2013) x Female | 1 | *Ref* |  |
|  | (Nov 2013 - May 2017) x Female | 0.87 | 0.79 - 0.95 | 0.002 |
|  | (Jun 2017 - Jul 2018) x Female | 0.57 | 0.47 - 0.68 | <0.001 |
| **Kasambya** | | | | |
| Diagnostic test done | B/S | 1 | *Ref* |  |
|  | RDT | 0.98 | 0.93 - 1.04 | 0.54 |
| Gender | Male | 1 | *Ref* |  |
|  | Female | 1.53 | 1.46 - 1.61 | <0.001 |
| Intervention period | Jan 2009 - Nov 2013 | 1 | *Ref* |  |
|  | Dec 2013 - Nov 2017 | 1.61 | 1.51 - 1.71 | <0.001 |
|  | Dec 2017 - Jul 2018 | 1.61 | 1.38 - 1.88 | <0.001 |
| Effect of gender by intervention period | (Jan 2009 - Nov 2013) x Female | 1 | *Ref* |  |
|  | (Dec 2013 - Nov 2017) x Female | 0.78 | 0.73 - 0.84 | <0.001 |
|  | (Dec 2017 - Jul 2018) x Female | 0.97 | 0.80 - 1.17 | 0.75 |
| **Aduku** | | | | |
| Diagnostic test done | B/S | 1 | *Ref* |  |
|  | RDT | 1.25 | 1.19 - 1.32 | <0.001 |
| Gender | Male | 1 | *Ref* |  |
|  | Female | 3.26 | 2.99 - 3.56 | <0.001 |
| Intervention period | Jan 2009 - Aug 2010 | 1 | *Ref* |  |
|  | Sep 2010 - Apr 2014 | 2.4 | 2.19 - 2.63 | <0.001 |
|  | May 2014 - May 2017 | 2.19 | 2.00 - 2.39 | <0.001 |
|  | Jun 2017 - Jul 2018 | 4.38 | 3.77 - 5.09 | <0.001 |
| Effect of gender by intervention period | (Jan 2009 - Aug 2010) x Female | 1 | *Ref* |  |
|  | (Sep 2010 - Apr 2014) x Female | 0.89 | 0.79 - 0.99 | 0.038 |
|  | (May 2014 - May 2017) x Female | 0.72 | 0.65 - 0.80 | <0.001 |
|  | (Jun 2017 - Jul 2018) x Female | 0.63 | 0.53 - 0.76 | <0.001 |
| **Nagongera** | | | | |
| Diagnostic test done | B/S | 1 | *Ref* |  |
|  | RDT | 1.25 | 1.16 - 1.34 | <0.001 |
| Gender | Male | 1 | *Ref* |  |
|  | Female | 2.29 | 2.18 - 2.41 | <0.001 |
| Intervention period | Jan 2009 - Nov 2013 | 1 | *Ref* |  |
|  | Dec 2013 - Jan 2015 | 1.19 | 1.07 - 1.33 | 0.001 |
|  | Feb 2015 - Jul 2018 | 2.35 | 2.13 - 2.60 | <0.001 |
| Effect of gender by intervention period | (Jan 2009 - Nov 2013) x Female | 1 | *Ref* |  |
|  | (Dec 2013 - Jan 2015) x Female | 1 | 0.87 - 1.15 | 0.995 |
|  | (Feb 2015 - Jul 2018) x Female | 0.79 | 0.70 - 0.89 | <0.001 |

Results in Table S2 showed that after accounting intervention period and for the effect of gender, the same being modified by intervention periods at all sites, diagnostic test used was only significantly associated with age category of confirmed malaria cases in Aduku and Nagongera but not in Walukuba or Kasambya. Importantly however, the effect of gender across all sites is seen to significantly increase in males, given its reduction in females by intervention periods relative to the baseline.

**Consideration of possible effect of changes in diagnostic testing methods**

Table S4. Association between age (in three categories) and covariates of interest among malaria confirmed cases, fitting an interaction between diagnostic test used (B/S vs. RDT) and intervention duration

| Factor | Categories | Coefficient | 95% CI | P - Value |
| --- | --- | --- | --- | --- |
| **Walukuba** | | | | |
| Gender | Male | 1 | *Ref* |  |
|  | Female | 1.34 | 1.29 - 1.39 | <0.001 |
| Malaria test done | Microscopy | 1 | *Ref* |  |
|  | RDT | 2.11 | 0.61 - 7.26 | 0.237 |
| Intervention period | Jan 2009 - Oct 2013 | 1 | *Ref* |  |
|  | Nov 2013 - May 2017 | 1.55 | 1.48 - 1.62 | <0.001 |
|  | Jun 2017 - Jul 2018 | 2.27 | 2.05 - 2.51 | <0.001 |
| Interaction term | (Jan 2009 - Oct 2013) x RDT | 1 | *Ref* |  |
|  | (Nov 2013 - May 2017) x RDT | 0.45 | 0.13 - 1.56 | 0.208 |
|  | (Jun 2017 - Jul 2018) x RDT | 0.56 | 0.16 - 1.98 | 0.372 |
| **Kasambya** | | | | |
| Gender | Male | 1 | *Ref* |  |
|  | Female | 1.4 | 1.35 - 1.45 | <0.001 |
| Malaria test done | Microscopy | 1 | Ref |  |
|  | RDT | 1.08 | 0.99 - 1.18 | 0.101 |
| Intervention period | Jan 2009 - Nov 2013 | 1 | *Ref* |  |
|  | Dec 2013 - Nov 2017 | 1.41 | 1.36 - 1.47 | <0.001 |
|  | Dec 2017 - Jul 2018 | 1.79 | 1.46 - 2.20 | <0.001 |
| Interaction term | (Jan 2009 - Nov 2013) x RDT | 1 | *Ref* |  |
|  | (Dec 2013 - Nov 2017) x RDT | 0.87 | 0.78 - 0.97 | 0.013 |
|  | (Dec 2017 - Jul 2018) x RDT | 0.79 | 0.62 - 1.00 | 0.055 |
| **Aduku** | | | | |
| Gender | Male | 1 | Ref |  |
|  | Female | 2.64 | 2.54 - 2.75 | <0.001 |
| Malaria test done | Microscopy | 1 | Ref |  |
|  | RDT | 1.05 | 0.87 - 1.28 | 0.603 |
| Intervention period | Jan 2009 - Aug 2010 | 1 | Ref |  |
|  | Sep 2010 - Apr 2014 | 2.2 | 2.08 - 2.33 | <0.001 |
|  | May 2014 - May 2017 | 1.79 | 1.69 - 1.90 | <0.001 |
|  | Jun 2017 - Jul 2018 | 3.77 | 3.15 - 4.52 | <0.001 |
| Interaction term | (Jan 2009 - Aug 2010) x RDT | 1 | Ref |  |
|  | (Sep 2010 - Apr 2014) x RDT | 1.46 | 1.13 - 1.87 | 0.003 |
|  | (May 2014 - May 2017) x RDT | 1.18 | 0.96 - 1.44 | 0.121 |
|  | (Jun 2017 - Jul 2018) x RDT | omitted | N/A |  |
| **Nagongera** | | | | |
| Gender | Male | 1 | Ref |  |
|  | Female | 2.18 | 2.09 - 2.28 | <0.001 |
| Malaria test done | Microscopy | 1 | Ref |  |
|  | RDT | 1.03 | 0.91 - 1.16 | 0.649 |
| Intervention period | Jan 2009 - Nov 2013 | 1 | Ref |  |
|  | Dec 2013 - Jan 2015 | 1.15 | 1.08 - 1.24 | <0.001 |
|  | Feb 2015 - Jul 2018 | 1.92 | 1.77 - 2.08 | <0.001 |
| Interaction term | (Jan 2009 - Nov 2013) x RDT | 1 | Ref |  |
|  | (Dec 2013 - Jan 2015) x RDT | 1.48 | 1.17 - 1.86 | 0.001 |
|  | (Feb 2015 - Jul 2018) x RDT | 1.35 | 1.14 - 1.59 | <0.001 |

Whereas diagnostic testing increasingly (in the last three years of the study) included RDT use, with the highest increase observed in Kasambya, Aduku, and Nagongera and least in Walukuba (Figure S4), the potential impact of this change in diagnostic testing method did not generally change the effect identified as due to control intervention activities. The significant interaction in Kasambya and Nagongera provides some evidence of an effect of change in diagnostic testing approach, however, after accounting for this effect, the impact of control interventions on age distribution of confirmed malaria cases persists and remains strongly statistically significant (Table S2). This, therefore, provides further evidence that given other factors at play, the upward shift from younger to older age-groups of malaria cases following successful malaria control interventions is significantly attributable to impacts of control interventions on malaria transmission.
